# Vanadium Toxicological Potential versus Its Pharmacological Activity: New Developments and Research

**DOI:** 10.1155/2016/7612347

**Published:** 2016-08-29

**Authors:** Agnieszka Ścibior, Juan Llopis, Alvin A. Holder, Mario Altamirano-Lozano

**Affiliations:** ^1^Laboratory of Oxidative Stress, Centre for Interdisciplinary Research, The John Paul II Catholic University of Lublin, 20-708 Lublin, Poland; ^2^Institute of Nutrition and Food Technology, Biomedical Research Center, University of Granada, 18071 Granada, Spain; ^3^Department of Chemistry and Biochemistry, Old Dominion University, Norfolk, VA 23529-0126, USA; ^4^Genetic and Environmental Toxicology Research Unit, FES-Zaragoza, UMIEZ Campus II, National Autonomous University of Mexico, 09230 México City, Mexico

Vanadium, which arouses interest of many research centers worldwide, is a biologically essential redox-active metal with an ability to evoke diametrically opposite effects. Due to its dual character and multidirectional action, vanadium receives a great deal of attention of pharmacologists and researchers from different scientific disciplines.

Despite the fact that extensive knowledge about vanadium has been gathered, many aspects of its action still require to be clearly defined. At present, there is a lack of sufficient data about the mechanisms of absorption, transport, and excretion of this metal. Relatively little is also known about the consequences and mechanisms of mutual interactions of vanadium with other elements, particularly those that have antioxidant potential. The question of interactions between elements, which may take place at different levels and can be used to elucidate the cellular mechanisms of response to combinations of metals, is always up to date and still an important issue in toxicology. The essentiality of vanadium to humans and the mechanisms of its toxic action also need to be elucidated. The above-mentioned aspects and likewise the pharmacological activity of vanadium, which raises hopes for use thereof in the treatment of certain diseases in humans, including diabetes, osteoporosis, and certain types of cancer as well as parasitic and infectious diseases, need to be further studied in order to fully understand and elucidate the toxicology as well as the biological activity and pharmacological potential of this element. More details in this research field may help to estimate the balance between adverse effects of vanadium (which has a relatively narrow margin of safety) and its potential therapeutic properties and to determine better its toxicity and therapeutic intervals which, in turn, allows us to devise and develop possible procedures of treatment of certain illnesses.

In this special issue, an attempt has been made to include reports that update our knowledge about the role of vanadium in toxicological processes and pharmacological applications and identify gaps in this research field, which would ensure better understanding of the specific behavior of this element. The special issue on vanadium compiles six (6) excellent manuscripts including reviews and research articles, which provide current and comprehensive knowledge about some aspects of the action of this metal.

The review article by M. Aureliano covers recent advances in the understanding of decavanadate toxicology and pharmacological applications. The Author (a) discusses in depth the modes of action of decavanadate (V_10_) through oxidative stress, effects on mitochondria, sarcoplasmic reticulum, and cytoskeleton, (b) provides information about some aspects of action of decavanadate (V_10_) and vanadate (V_1_), and (c) highlights the significance of understanding the V_10_ toxicology and pharmacological activities as important targets to elucidate the biological activities of several polyoxometalates in order to make them available and safe for clinical use. In turn, the review article by Tsave et al. focuses on the role of vanadium in cellular and molecular immunology. The Authors nicely and concisely illustrate (a) the effects of selected vanadium species in the immune system processes, (b) forms of vanadium exhibiting immunogenic activity, and (c) the key role(s) of vanadium in promoting innate and adaptive immunity as well as (d) current obstacles to be overcome by specifically designed vanadium metallodrugs in cancer immunotherapeutics. The research data collected in this review highlight the synthetic and structural bioinorganic profile of vanadium along with its biological activity attributes, collectively formulating the significant potential of unique structure-based and immune process-specific vanadodrugs for the detection, prevention, and treatment of immune system aberrations.

As far as the research articles are concerned, the report of Treviño et al. presents metforminium decavanadate (MetfDeca) as a potential metallopharmaceutical drug for the treatment of diabetes mellitus. The Authors showed efficiency of MetfDeca in improving serum profiles of carbohydrates and lipids and revealed a protective effect of MetfDeca on pancreatic beta cells of rats with model type 1 diabetes mellitus. In turn, the research article of García-Rodrígez et al. illustrates the* in vivo* effects of vanadium (as vanadium pentoxide) and certain antioxidants (ascorbic acid and alpha-tocopherol) on apoptotic, cytotoxic, and genotoxic activity in mice. The Authors demonstrated that both these antioxidants were able to protect cells against vanadium pentoxide-induced genetic damage. The research article of Christensen et al., in turn, presents the effectiveness of bisphosphonate and vanadium-bisphosphonate compounds against axenic* Leishmania tarentolae*. Specifically, the Authors showed that two polyoxometalates (POMs) with nitrogen containing bisphosphonate ligands: vanadium/alendronate [V_5_(Ale)_2_] and vanadium/zoledronate [V_3_(Zol)_3_] complexes were effective in inhibiting the growth of* L. tarentolae* and suggested that V_3_(Zol)_3_ may be effective in a skin cream formulation as a weekly or daily treatment for cutaneous leishmaniasis. Finally, the report of Folarin et al. describes the effects of administration of vanadium on memory in mice. The Authors indicated that mice exposed to vanadium exhibited no difference in learning abilities but had significant loss in memory acumen after 3 months of exposure. They also revealed that the memory deficit induced by chronic administration of vanadium in mice is reversible, but only after a long period of vanadium withdrawal.

We believe that the information provided in this special issue will be of interest to readers who are interested in vanadium and to those of Oxidative Medicine and Cellular Longevity in general.

